# Body Mass Index and Decline of Cognitive Function

**DOI:** 10.1371/journal.pone.0148908

**Published:** 2016-02-11

**Authors:** Sujin Kim, Yongjoo Kim, Sang Min Park

**Affiliations:** 1 Takemi Program in International Health, Harvard T. H. Chan School of Public Health, Boston, Massachusetts, United States of America; 2 Department of Social and Behavioral Sciences, Harvard T. H. Chan School of Public Health, Boston, Massachusetts, United States of America; 3 Department of Family Medicine & Department of Biomedical Sciences, Seoul National University College of Medicine, Seoul, South Korea; University of Missouri, UNITED STATES

## Abstract

**Background:**

The association between body mass index (BMI) and cognitive function is a public health issue. This study investigated the relationship between obesity and cognitive impairment which was assessed by the Korean version of the Mini-mental state examination (K-MMSE) among mid- and old-aged people in South Korea.

**Methods:**

A cohort of 5,125 adults, age 45 or older with normal cognitive function (K-MMSE≥24) at baseline (2006), was derived from the Korean Longitudinal Study of Aging (KLoSA) 2006~2012. The association between baseline BMI and risk of cognitive impairment was assessed using multiple logistic regression models. We also assessed baseline BMI and change of cognitive function over the 6-year follow-up using multiple linear regressions.

**Results:**

During the follow-up, 358 cases of severe cognitive impairment were identified. Those with baseline BMI≥25 kg/m^2^ than normal-weight (18.5≤BMI<23 kg/m^2^) were marginally less likely to experience the development of severe cognitive impairment (adjusted odds ratio [aOR] = 0.73, 95% CI = 0.52 to 1.03; *P*_trend_ = 0.03). This relationship was stronger among female (aOR = 0.63, 95% CI = 0.40 to 1.00; *P*_trend_ = 0.01) and participants with low-normal K-MMSE score (MMSE: 24–26) at baseline (aOR = 0.59, 95% CI = 0.35 to 0.98; *P*_trend_<0.01). In addition, a slower decline of cognitive function was observed in obese individuals than those with normal weight, especially among women and those with low-normal K-MMSE score at baseline.

**Conclusion:**

In this nationally representative study, we found that obesity was associated with lower risk of cognitive decline among mid- and old-age population.

## Introduction

Overweight and obese individuals are known to be at a higher risk for dementia [1–8]. Adiposity may be directly and indirectly, by increasing other vascular risk factors, linked to cognitive impairment and dementia [9]. Nevertheless, the association between body mass index (BMI) and risk of dementia is far from clear. In late life, the detrimental consequences of being obese or overweight appear less apparent, and there are even potential protective effects. For example, individuals with high BMI (BMI≥25 kg/m^2^) have been observed to have a lower risk of cognitive impairment [10–16]. Furthermore, a recent study based on a large population, almost two million, reports an inverse association between BMI and the risk of dementia in both mid- and late-life [17]. There is continuing controversy regarding the protective effects or detrimental effects of a high BMI.

The number of reported cases of dementia is rapidly increasing in Asia. By 2050, the prevalence will increase three times, and more than half of people worldwide living with dementia will live in the Asia Pacific region [18]. In particular, as Asians tend to have higher amounts of abdominal fat at lower BMIs [19], an alternative definition of overweight (BMI 23.0–24.9 kg/m^2^) and obesity (BMI≥25 kg/m^2^) has been proposed for Asian populations [20]. For example, mortality risks significantly increased at BMI≥25 kg/m^2^, rather than at BMI≥30 kg/m^2^ [21]. In addition, an examination of racial and ethnic differences among older adults found that the positive association of obesity with cognitive performance appeared only in Whites, and there were negative associations in Hispanics and Blacks [22]. However, while most of the existing literature has been based on western countries, the causal role of BMI as a risk factor for cognitive impairment among middle-and old-age individuals in Asian countries has rarely been investigated. Although one particular study from Asia found that obese adults experienced slower cognitive decline, it was quite small (based on fewer than 800 subjects) and short in duration of follow-up (2 years) [23]. Thus, it is unclear whether or not there is a protective association between high BMI and cognitive impairment in Asian populations.

Meanwhile, some factors may affect associations between BMI and cognitive function. For example, vascular risk factors are related to both dementia and depression [24]. Individuals with depression experience faster cognitive decline and high risk of dementia, [25–28] and also have a greater risk of vascular and neurological outcomes [29, 30], myocardial infarction and coronary heart disease [31, 32], and stroke [33, 34]. In addition, a meta-analysis showed a reciprocal positive association between obesity and depression [35] while obesity is known as a risk factor for neurobiological diseases like stroke [36, 37]. Since there might be interactions between risk factors, it could be worthwhile to examine whether the relations of obesity to cognitive function depends on other factors.

More research would be needed to better understand the relationships between obesity and cognitive impairment in Asian countries. This research investigated how BMI relates to cognitive impairment/decline in middle- and old-aged people with healthy cognitive function, using a nationally representative longitudinal sample of South Korean middle and old-aged adults.

## Methods

### Study population and source of data

**Fig 1:**
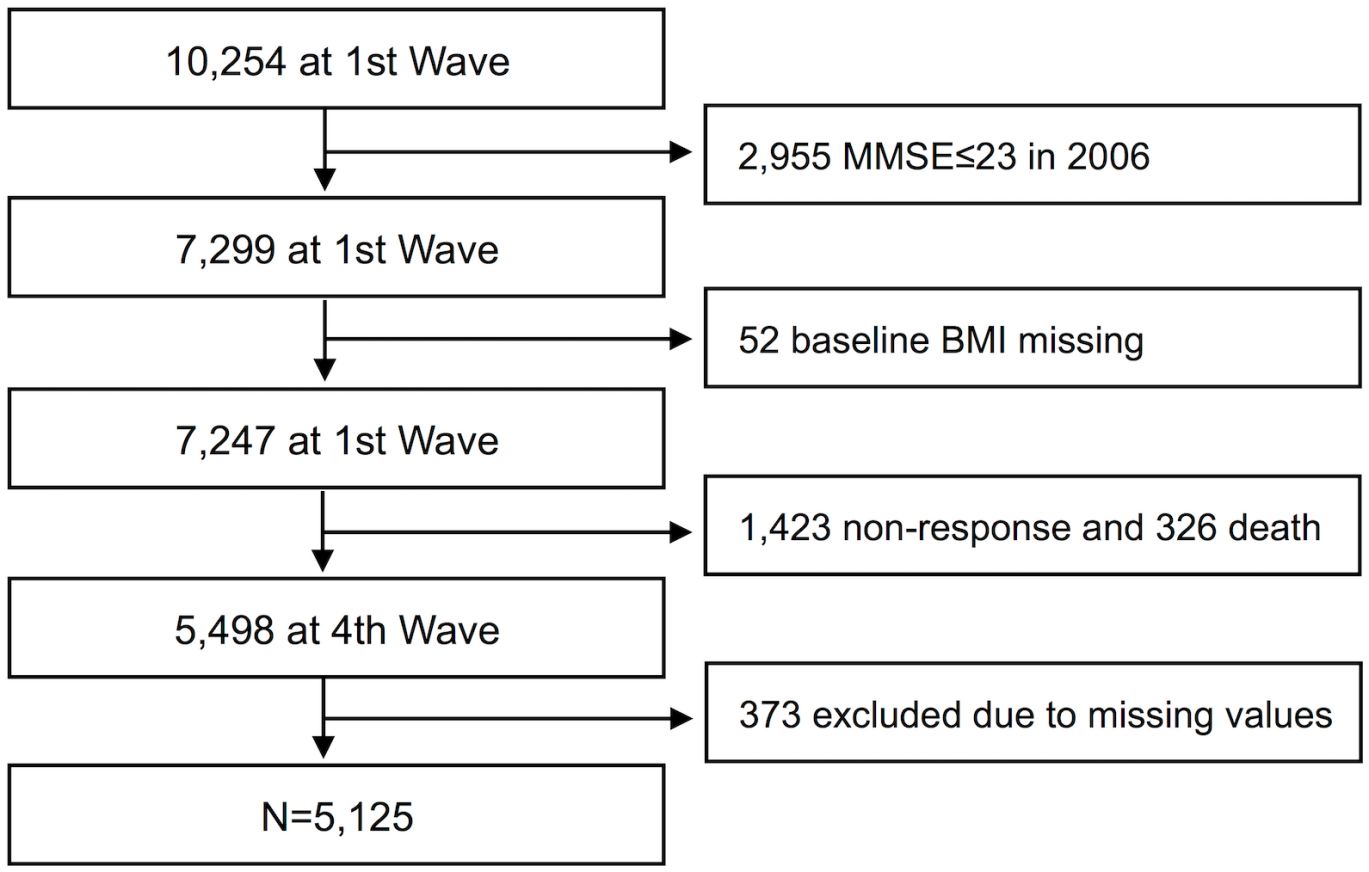
Schematic diagram of the study population.

The present study used data from the Korean Longitudinal Study of Aging (KLoSA), which is a nationally representative panel survey on middle- and old-aged populations (over 45), administered, since 2006, by the Korea Labor Institute [38]. A total of 6,171 households were sampled from 1,000 sample enumeration districts. Through the computer assisted personal interviewing procedure, data on demographics, family structure, health status, medical history, income and employment status for 10,254 individuals aged 45 years or older were collected in 2006 and followed-up with for every even-numbered year. Of four publicly available waves (2006, 8, 10, and 12), we used the first and fourth waves. Of the 10,254 eligible population, for whom cognitive function is measured at baseline, 7,299 individuals with normal cognitive function (the Korean version of the Mini-mental state examination, K-MMSE>23) were defined as the baseline study population [39]. 52 participants with missing values for weight and height were excluded from the first wave, and 2,122 individuals were additionally excluded from the fourth wave (326 due to death, 1,423 due to non-response, and 373 due to missing values for relevant variables). Therefore, we set our analytic sample as the remaining 5,125 respondents (Fig 1).

### Measurements

The study considered a twofold dependent variable, which was measured by using the K-MMSE.: 1) onset of severe cognitive impairment; and 2) the extent of decline in cognitive functions over a six-year follow-up period. The K-MMSE included 11 items in 7 categories of cognitive functions, including orientation for time and place, registration, attention & calculation, recall, language, and visual construction [39, 40]. The total score of the measure ranges from 0 to 30; the higher the score, the better the cognitive function. The validity of the K-MMSE was reported elsewhere [40]. We followed the conventional classification criteria for cognitive function, categorizing K-MMSE scores as severe cognitive impairment (SCI, K-MMSE≤17), mild cognitive impairment (MCI, 18≤K-MMSE≤23), and normal cognitive function (K-MMSE≥24) [39, 40].

BMI (kg/m^2^) was estimated from self-reported weight and height in 2006, and was classified as underweight (BMI<18.5), normal (18.5≤BMI<23), overweight (23≤BMI<25), and obese (BMI≥25) according to the revised Asia-Pacific BMI criteria by the World Health Organization Western Pacific Region [20]. A validation study of the self-reported measure using a subsample (N = 510) of KLoSA reported that there was an adequate degree of correlation between self-reported and measured BMI values (Pearson’s correlation coefficient 0.837 for men, and 0.865 for women) [41].

From the first wave, other covariates were collected: age (45–54, 55–64, ≥65 years), sex, marital status (married, unmarried), education (elementary, middle, high school, and ≥college), equivalized household income (quartile), insurance status (Medicaid vs National Health Insurance), residence (urban, rural), cigarette smoking (yes, no), alcohol consumption (yes, no), regular physical activity (more than once a week, no), activities of daily livings (0, ≥1), depression (the Center for Epidemiologic Studies-Depression 10-item Scale≥4, <4), and comorbidity (at least one of hypertension, diabetes, cardiovascular disease, and/or cerebrovascular disease, 0).

### Statistical analysis

To investigate the association between BMI and cognitive impairment, we used two approaches in framing the outcome: 1) new-onset of SCI after 6 years’ follow-up as a binary outcome (K-MMSE≤17), and 2) cognitive decline on a continuous scale by using the change in K-MMSE scores from 2006 to 2012. As our primary approach, for the association between baseline body weight status and new-onset of SCI, we performed multiple logistic regressions for the selected analytic sample and estimated adjusted Odds Ratios (aORs). Additionally, the association between baseline body weight status and cognitive decline (change in K-MMSE scores) was assessed using multiple linear regression, and adjusted means of change were estimated. For both logistic and linear models, sets of covariates were sequentially included as follows: Model 1 (age, gender), Model 2 (marital status, health insurance, income, education, place of living, based on Model 1), Model 3 (physical activity, drinking, and smoking, based on Model 2), and Model 4 (comorbidity, activities of daily livings, depression, and baseline K-MMSE). We set Model 4 as the final model for both outcomes.

After investigating the overall impact of baseline BMI on change in cognitive function at follow-up, we performed stratified analyses by age, gender, baseline K-MMSE score (24–26, ≥27), smoking, drinking, physical activity, depression and comorbidity status in order to figure out the potentially differential impact of weight status on cognitive decline across the subgroups. We applied longitudinal sampling weight, and adjusted for complex survey design by accounting for strata and cluster. For all analyses, we used Stata ver 12.0 and set the level of significance as 0.05 (two-sided).

The study was conducted in accordance with the Ethical Principles for Medical Research Involving Human Subjects, as defined by the Helsinki Declaration. Participants were required to read and sign an agreement form before participating in the KLoSA study. For the present study, the Institutional Review Board of Seoul National University Hospital (South Korea) waved institutional review board approval because this study used only publicly available de-identified data.

## Results

Table 1 presented the baseline characteristics of the study population. Among the total of 5,125 subjects, 24.6% were obese, 31.5% overweight, 41.7% normal weight, and 2.2% underweight. Across the age groups (45–54, 55–64, 65+), the oldest group (age≥65) had the smallest proportions of obese (22.7%) and overweight (30.9%) individuals, while the group had the largest proportion of underweight (3.2%) and normal-weight (43.2%) individuals. Interestingly, the proportions of obese/overweight individuals were greater among those who had regular physical activity (26.9% for obese, 32.2% for overweight) than those who did not (22.8% for obese, 30.9% for overweight). Furthermore, the proportions of obese/overweight individuals were significantly greater among those who had chronic disease (35.5% vs. 20.5% for obese, 32.4% vs. 31.1% for overweight) and were depressed (24.8% vs. 24.0% for obese, 32.6% vs. 27.4% for overweight).

**Table 1:** General characteristics of study population.

	Overall		BMI<18.5		18.5~23		23~25		25+		p-value
	n = Weighted%		n = Weighted%		n = Weighted%		n = Weighted%		n = Weighted%		
General Information	5,125	100.0	116	2.2	2,169	41.7	1,599	31.5	1,241	24.6	
Age (years)											
45–54	2,127	55.9	36	1.8	936	42.6	642	31.1	513	24.5	0.06
55–64	1,674	28.6	38	2.5	654	39.1	554	32.5	428	25.9	
65+	1,324	15.6	42	3.2	579	43.2	403	30.9	300	22.7	
Sex											
Male	2,515	51.3	56	1.9	1,034	39.6	840	34.3	585	24.1	<0.01
Female	2,610	48.7	60	2.5	1,135	43.9	759	28.5	656	25.2	
Marital status											
Married	4,492	88.5	91	2.0	1,919	42.0	1,397	31.4	1085	24.6	0.08
Unmarried	633	11.5	25	3.8	250	39.8	202	31.7	156	24.7	
Education											
Elementary School	399	5.7	19	5.1	184	44.8	104	25.5	92	24.7	<0.01
Middle School	1,320	21.8	33	2.3	528	39.6	397	30.2	362	28.0	
High School	1,011	19.8	19	1.9	405	39.6	336	32.5	251	26.0	
College+	2,395	52.7	45	2.0	1,052	43.1	762	32.2	536	22.7	
Household income											
1Q	1,305	22.4	52	4.0	552	41.6	399	31.4	302	23.1	<0.01
2Q	1,262	23.5	21	1.7	523	40.1	375	29.9	343	28.3	
3Q	1,391	28.4	26	2.0	563	39.7	479	35.1	323	23.2	
4Q	1,167	25.7	17	1.4	531	45.5	346	29.0	273	24.2	
Insurance											
Medicaid	203	3.9	9	3.7	80	40.9	56	27.1	58	28.4	0.33
National Health Insurance	4,922	96.1	107	2.2	2,089	41.7	1,543	31.7	1183	24.5	
Location											
Urban	3,943	79.9	89	2.2	1,615	40.7	1,259	31.9	980	25.2	0.06
Rural	1,182	20.1	27	2.1	554	45.8	340	29.6	261	22.4	
Cigarette smoking											
No	3,505	66.5	72	2.3	1,488	42.2	1,065	30.2	880	25.3	0.09
Yes	1,620	33.5	44	2.1	681	40.7	534	34.0	361	23.2	
Alcohol consumption											
No	2,909	52.7	71	2.4	1,245	42.6	884	30.2	709	24.9	0.27
Yes	2,216	47.3	45	2.0	924	40.7	715	32.9	532	24.3	
Physical Activity											
Yes	2,262	43.9	35	1.4	914	39.4	724	32.2	589	26.9	<0.01
No	2,863	56.1	81	2.8	1,255	43.5	875	30.9	652	22.8	
Activities of daily livings											
0	5,084	99.3	116	2.2	2,152	41.7	1,589	31.5	1227	24.5	0.31
1+	41	0.7	0	0.0	17	36.5	10	25.4	14	38.1	
Depression^a^											
Yes	3,919	78.5	77	1.9	1,634	40.7	1,253	32.6	955	24.8	<0.01
No	1,206	21.5	39	3.3	535	45.3	346	27.4	286	24.0	
Comorbidity^b^											
0	3,555	72.6	85	2.3	1,676	46.1	1,085	31.1	709	20.5	<0.01
+1	1,570	27.4	31	2.0	493	30.1	514	32.4	532	35.5	

^a^ The Center for Epidemiologic Studies-Depression 10-item Scale≥4 (Yes), <4 (No).

^b^ Hypertension, diabetes, cardiovascular disease, or cerebrovascular diseases.

Tables 2 and 3 displayed the results from the multiple logistic regression models for the association between baseline BMI and new-onset of SCI after the six year follow-up. Overall, there was no statistically significant evidence that either being overweight or underweight in 2006 were associated with increased/decreased risk of SCI. However, though not statistically significant, when compared with normal weight, the point estimates of aORs for underweight (aOR = 1.44, 95% CI: 0.74, 2.68), overweight (aOR = 0.87, 95% CI: 0.66, 1.15), and obese (aOR = 0.73, 95% CI: 0.52, 1.03) categories showed a linear trend in the risk of SCI after the six year follow-up across weight status at baseline (p for trend = 0.03).

**Table 2:** Adjusted Odd Ratios of baseline body mass index for severe cognitive impairment in Korean adults 45+ years.

	Baseline body mass index				
	<18.5	18.5~23	23~25	25+	*p* for trend
All	(*n = 16*)	(*n = 166*)	(*n = 110*)	(*n = 66*)	
Model 1	1.55	1.00	0.87	0.78	0.05
(95% CI) ^a^	(0.85–2.83)		(0.66–1.15)	(0.56–1.09)	
Model 2	1.42	1.00	0.88	0.76	0.04
(95% CI) ^b^	(0.76–2.64)		(0.66–1.16)	(0.54–1.07)	
Model 3	1.42	1.00	0.88	0.76	0.04
(95% CI) ^c^	(0.76–2.65)		(0.66–1.15)	(0.54–1.06)	
Model 4	1.44	1.00	0.87	0.73	0.03
(95% CI) ^d^	(0.78–2.68)		(0.66–1.15)	(0.52–1.03)	
Men	(*n = 7*)	(*n = 79*)	(*n = 53*)	(*n = 33*)	
Model 1	1.27	1.00	0.84	0.86	0.38
(95% CI) ^a^	(0.54–2.98)		(0.56–1.28)	(0.53–1.39)	
Model 2	1.18	1.00	0.86	0.86	0.43
(95% CI) ^b^	(0.48–2.90)		(0.56–1.31)	(0.53–1.41)	
Model 3	1.18	1.00	0.86	0.86	0.43
(95% CI) ^c^	(0.49–2.87)		(0.57–1.31)	(0.52–1.41)	
Model 4	1.21	1.00	0.86	0.83	0.38
(95% CI) ^d^	(0.50–2.91)		(0.56–1.32)	(0.49–1.41)	
Women	(*n = 9*)	(*n = 87*)	(*n = 57*)	(*n = 33*)	
Model 1	1.88	1.00	0.87	0.68	0.03
(95% CI) ^a^	(0.84–4.20)		(0.60–1.25)	(0.43–1.06)	
Model 2	1.80	1.00	0.88	0.66	0.03
(95% CI) ^b^	(0.79–4.10)		(0.61–1.27)	(0.42–1.05)	
Model 3	1.74	1.00	0.89	0.66	0.03
(95% CI) ^c^	(0.75–4.04)		(0.61–1.28)	(0.42–1.05)	
Model 4	1.77	1.00	0.87	0.63*	0.01
(95% CI) ^d^	(0.77–4.05)		(0.60–1.27)	(0.40–1.00)	
Baseline K-MMSE:24–26	(*n = 6*)	(*n = 92*)	(*n = 67*)	(*n = 42*)	
Model 1	2.50*	1.00	0.71	0.71	0.02
(95% CI) ^a^	(1.07–5.88)		(0.46–1.09)	(0.43–1.16)	
Model 2	2.26	1.00	0.73	0.69	0.03
(95% CI) ^b^	(0.93–5.49)		(0.47–1.13)	(0.41–1.15)	
Model 3	2.14	1.00	0.72	0.69	0.03
(95% CI) ^c^	(0.87–5.29)		(0.46–1.12)	(0.41–1.16)	
Model 4	2.20	1.00	0.67	0.59*	<0.01
(95% CI) ^d^	(0.87–5.57)		(0.43–1.05)	(0.35–0.98)	
Baseline K-MMSE:27–30	(*n = 10*)	(*n = 74*)	(*n = 43*)	(*n = 24*)	
Model 1	0.96	1.00	0.97	0.82	0.38
(95% CI) ^a^	(0.39–2.35)		(0.69–1.37)	(0.54–1.25)	
Model 2	0.93	1.00	0.97	0.80	0.34
(95% CI) ^b^	(0.37–2.31)		(0.69–1.38)	(0.52–1.23)	
Model 3	0.94	1.00	0.97	0.79	0.30
(95% CI) ^c^	(0.38–2.35)		(0.68–1.37)	(0.52–1.21)	
Model 4	0.95	1.00	0.98	0.80	0.36
(95% CI) ^d^	(0.38–2.35)		(0.69–1.39)	(0.52–1.24)	

Abbreviations: K-MMSE, the Korean version of the Mini-mental state examination; CI, confidence interval.

* p < .05.

n = number of cases of severe cognitive impairment (K-MMSE ≤ 17 in 2012)

Baseline body mass index was estimated from the 2006 survey.

^a^ Adjusted for gender and age from the 2006 survey.

^b^ Based on model 1, model 2 was further adjusted for marital status, health insurance, income, educational level, and living place from the 2006 survey.

^c^ Based on model 2, model 3 was further adjusted for physical activity, drinking, and smoking status from the 2006 survey.

^d^ Based on model 3, model 4 was further adjusted for comorbidity, baseline K-MMSE score, activities of daily livings and depression from the 2006 survey.

**Table 3:** Adjusted Odd Ratios of baseline body mass index for severe cognitive impairment by demographic and behavioral factors in Korean adults 45+ years.

	Baseline body mass index				
	<18.5	18.5~23	23~25	>25	*p* for trend
Ever smoker	(*n = 5*)	(*n = 60*)	(*n = 25*)	(*n = 20*)	
adjusted OR	0.88	1.00	0.50*	0.62	0.06
(95% CI)	(0.29–2.66)		(0.29–0.87)	(0.33–1.16)	
Never smoker	(*n = 11*)	(*n = 106*)	(*n = 85*)	(*n = 46*)	
adjusted OR	1.90	1.00	1.11	0.79	0.14
(95% CI)	(0.94–3.86)		(0.80–1.55)	(0.53–1.19)	
Current drinking	(*n = 2*)	(*n = 70*)	(*n = 35*)	(*n = 23*)	
adjusted OR	0.46	1.00	0.65	0.49*	0.02
(95% CI)	(0.10–2.15)		(0.41–1.03)	(0.27–0.88)	
No drinking	(*n = 14)*	(*n = 96*)	(*n = 75*)	(*n = 43*)	
adjusted OR	2.47*	1.00	1.10	0.98	0.41
(95% CI)	(1.21–5.05)		(0.79–1.55)	(0.65–1.49)	
No physical activity	(*n = 11*)	(*n = 100)*	(*n = 69*)	(*n = 33*)	
adjusted OR	1.46	1.00	1.04	0.72	0.10
(95% CI)	(0.68–3.13)		(0.74–1.46)	(0.46–1.13)	
Physical activity	(*n = 5*)	(*n = 66*)	(*n = 41*)	(*n = 33*)	
adjusted OR	1.45	1.00	0.68	0.75	0.22
(95% CI)	(0.51–4.12)		(0.43–1.10)	(0.40–1.39)	
Age ≥ 65	(*n = 10*)	(*n = 86*)	(*n = 58*)	(*n = 28*)	
adjusted OR	2.02	1.00	0.94	0.56*	<0.01
(95% CI)	(0.86–4.75)		(0.64–1.38)	(0.35–0.92)	
Age <65	(*n = 6*)	(*n = 80*)	(*n = 52*)	(*n = 38*)	
adjusted OR	1.14	1.00	0.81	0.78	0.19
(95% CI)	(0.46–2.84)		(0.55–1.18)	(0.50–1.21)	
No depression	(*n = 7*)	(*n = 102*)	(*n = 71)*	(*n = 52*)	
adjusted OR	0.88	1.00	0.86	0.86	0.44
(95% CI)	(0.39–2.01)		(0.60–1.23)	(0.58–1.28)	
Depression	(*n = 9*)	(*n = 64*)	(*n = 39*)	(*n = 14*)	
adjusted OR	2.31	1.00	0.90	0.42*	<0.01
(95% CI)	(0.90–5.96)		(0.56–1.45)	(0.21–0.86)	
Comorbidity: 0	(*n = 11*)	(*n = 112*)	(*n = 63*)	(*n = 32*)	
adjusted OR	1.59	1.00	0.91	0.82	0.18
(95% CI)	(0.78–3.24)		(0.64–1.29)	(0.53–1.28)	
Comorbidity: 1+	(*n = 5*)	(*n = 54*)	(*n = 47*)	(*n = 34*)	
adjusted OR	1.15	1.00	0.80	0.60*	0.04
(95% CI)	(0.38–3.49)		(0.50–1.26)	(0.36–1.00)	

Abbreviations: K-MMSE, the Korean version of the Mini-mental state examination; OR, Odds Ratio; CI, confidence interval.

* p < .05.

n = number of cases of severe cognitive impairment (K-MMSE ≤ 17 in 2012).

Baseline body mass index was estimated from the 2006 survey. Age, gender, marital status, health insurance, income, educational level, living place, physical activity, smoking status, drinking status, comorbidity, activities of daily livings, depression, and baseline K-MMSE score from the 2006 survey were adjusted for.

A significant association between weight status and the new-onset of SCI was found in some subgroups. When stratified by gender, women with obesity in 2006 showed significantly lower risk of SCI (aOR = 0.63, 95% CI: 0.40, 1.00) compared with women with normal weight. Though statistically not significant, the directionalities of point estimates of aORs among each gender group were consistent with the result from overall analysis. Particularly, the linear trend in the association between weight status and risk of SCI was more apparent among women (p for trend = 0.01) than among men (p for trend = 0.38). Among those with lower normal cognitive function (24≤K-MMSE≤26), obesity was associated with lower risk of SCI when compared to normal weight (aOR = 0.59, 95% CI: 0.35, 0.98) with notable linear trend of the risk of SCI across weight status (p for trend <0.01) (Table 2).

When looking at the results from the subgroup analyses in Table 3, among older adults (age≥65), obesity at baseline was associated with lower risk of SCI at 6 years’ follow-up and a significant linear trend in the association between weight status and the new-onset of SCI (p = 0.004). When stratified by drinking status, current drinkers with obesity showed significantly smaller risk of SCI, compared to current drinkers with normal weight (aOR = 0.49, 95% CI: 0.27, 0.88). In addition, currently non-drinking status paired with underweight status was associated with a greater risk of SCI, compared to non-drinking paired with normal weight (aOR = 2.47, 95% CI: 1.21, 5.05).

**Fig 2:**
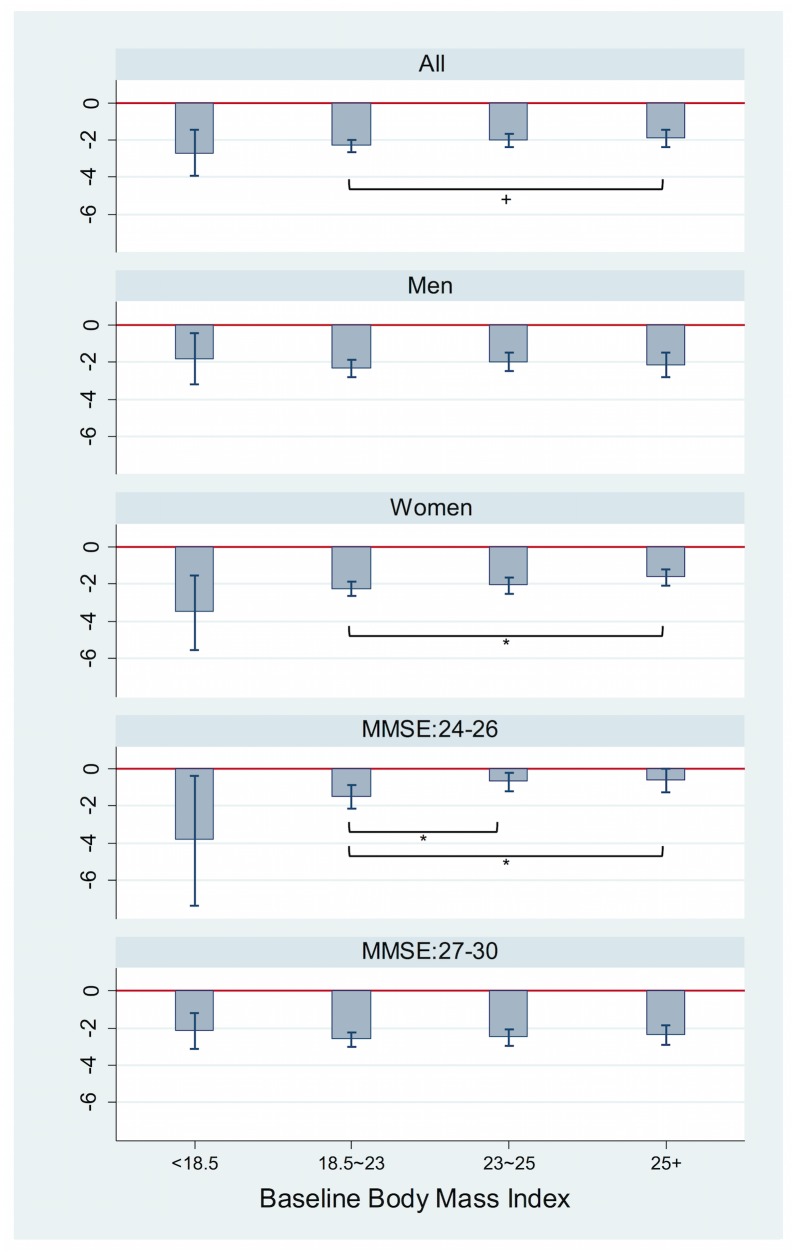
Adjusted mean of change of K-MMSE score across baseline body mass index in Korean adults 45+ years. Abbreviations: K-MMSE, the Korean version of the Mini-mental state examination. Reference category: body mass index 18.5–23 kg/m^2^. * p < .05 ** p < .01, *** p < .001. Change of K-MMSE score was calculated by subtracting 2006 K-MMSE from 2012 K-MMSE. Baseline BMI was measured from the 2006 survey. Age, gender, marital status, health insurance, income, educational level, living place, physical activity, smoking and drinking status, comorbidity, activities of daily livings, depression, and baseline K-MMSE score from the 2006 survey were adjusted for.

Fig 2 presented the results from the multiple linear regression models for the association between baseline BMI and change in cognitive function as a continuous scale over the six-year follow-up. Among all individuals, being obese at baseline appeared to be related to a slower decline in cognitive function over the following six years, compared to individuals with normal weight. When stratified by gender, women with obesity showed, on average, a smaller decrease in K-MMSE score (beta = 0.6, 95% CI: 0.04, 1.16, p = 0.04) than women with normal weight at baseline. Among individuals with lower normal cognitive function (24≤K-MMSE≤26) at baseline, compared with higher normal cognitive function, being overweight and obese showed a significantly smaller cognitive decline (beta for overweight = 0.80, 95% CI: 0.02, 1.58, p = 0.04; beta for obesity = 0.88, 95% CI: 0.06, 1.69, p = 0.04) (Fig 2).

## Discussion

In this nationally representative study, we provide the prospective investigation of BMI and decline of cognitive function. Our results suggest that being obese (BMI≥25 kg/m^2^), especially in late-life, is related to a lower risk of cognitive impairment, compared with having a normal weight (18.5≤BMI<23 kg/m^2^). This relationship became stronger when we adjusted for confounding factors, such as health behavior and health status. We also found that, although cognitive function decreased over the follow-up period, obese individuals, as compared to those with normal weight, experienced a slower decline in cognitive function. In addition, the protective association of high BMI with cognitive performance was more prominent in women and individuals with relatively low-normal K-MMSE score at baseline.

The protective association of obesity (BMI≥25 kg/m^2^) with cognitive function observed in the present study is broadly consistent with previous studies in western countries. Whereas positive association between obesity and cognitive impairment is generally expected, protective effects, rather than detrimental consequences, of a high BMI on cognitive function has often been observed, especially during old age. Older persons with high BMI scores (i.e. BMI≥25) at baseline have less risk of dementia [1–8, 15, 42], and experience slower declines in cognitive function [12]. In addition, Qizilbash and colleagues [17] found inverse associations between BMI and dementia, even in mid-life, based on almost 2 million primary care patients in the UK. Another study analyzing data from the Whitehall study replicated the same results [43]. Our results showed the protective association of high BMI with cognitive impairment among adults 65 years and older. When the sample was restricted to adults 45–65 years, the protective association was not statistically significant, and the protective relationship between high BMI and cognitive function became more evident among adults age 65 or older. Although weight loss that seemingly begins years before the onset of clinical syndrome of dementia may be related to these findings [44], it would not completely account for those associations. For instance, according to a previous study, individuals whose BMI was high (25–30) over the past four years experienced lower cognitive impairment than those with consistent normal range (20–25) of BMI [11]. Furthermore, a study with a 26-year follow-up period reported that high late-life BMI was associated with lower risk of Alzheimer’s disease (0.89, 0.81–0.98) and marginally significant association with lower onset of dementia [7]. That is, although being underweight and losing weight may be an early marker of cognitive impairment [7, 11, 44], high BMI scores during old age tend to have consistently a protective association with cognitive function.

Meanwhile, in the present study, being underweight (BMI<18.5) rarely shows statistically significant associations with the development of cognitive impairment, contrary to previous findings. This is probably due to the fact that in middle- and old-age populations, being underweight has a high mortality risk [45], so cognitive impairment is possibly underestimated in the group. In addition, this study restricted the sample population to individuals with a normal range of K-MMSE scores at baseline, which in turn led the group of underweight individuals to lose more samples than other groups. Indeed, the number of underweight individuals at baseline was the smaller (n = 116, 2.2%) than the other weight groups.

Relationships between high BMI and cognitive impairment have been explained since adiposity is directly related to hyperinsulinemia, adipokines and cytokines, and indirectly related to vascular risk factors [9]. It is plausible that the role of BMI in dementia may change over the course of life. That is, the cut-off distinguishing underweight from normal conditions may differ in the elderly and younger populations. For example, for older people, similarly to the protective association of high BMI with cognitive function observed, the detrimental effects of high BMI on mortality are attenuated with increasing age [46], and a BMI lower than 25 correlates with a higher risk of death [47]. In addition, previous studies suggest that a BMI of less than 25 may be indicative of malnutrition for older people while nutritional and cognitive status correlates closely via the fat brain axis [48, 49].

The protective associations of high BMI with cognitive impairment might be related to changes in body composition. Aging is characterized by the loss of lean body mass [46], and higher lean body mass may be involved in reducing the risk of cognitive impairment in an older population [50]. Furthermore, high BMI may also result from increased accumulation of fat in regions other than the abdominal area. Larger leg fat mass in older individuals has been associated with improved glucose metabolism [51], which could potentially contribute to a lower risk of development of cognitive impairment. In addition, the association might be mediated by serum urate, which is positively correlated with BMI and, by acting as antioxidants in the brain, might prohibit development of neurodegenerative disease [52, 53]. Further biomarker studies are needed to clarify the relationship and mechanisms.

The present study has several limitations. First, obesity was assessed based on self-reported body weight and height in KLoSA, which can lead to biased estimates. However, the validation study reported adequate degree of correlation between self-reported and measured BMI data (Pearson’s correlation: 0.84 for men, and 0.87 for women) [41]. In addition, in our modeling approach, we adjusted factors that were associated with the discrepancy in BMI measures from the validation study, including region, age, and educational level, as well as other covariates such as chronic disease histories and activities of daily livings. Second, the disagreement in BMI measures may be associated with cognitive function, which could bias the findings of the present study. However, we excluded participants with lower than normal range of K-MMSE score at baseline. Third, selection bias due to differential loss to follow-up can be an issue. Among 7,247 participants with normal cognitive function in 2006, 29% were not included in the analysis due to death (n = 326), non-responses due to reasons other than death (n = 1,423), and missing values (n = 373). However, the mean values of K-MMSE score at baseline were similar between the study population (mean K-MMSE score = 27.36 for N = 5,125) and those who dropped out (mean score = 27.42 for N = 2,122) over the follow-up. Therefore, it is possible that the potential issue of attrition of this study might not have influenced the findings of the present study. Lastly, the residuals of linear regressions for MMSE change did not appear normally distributed when the Kolmogorov Smirnov test were used. Although analysis based on log-transformation showed consistent results (i.e., There were the significantly positive associations of obesity with MMSE change among women and adults with low-normal K-MMSE score at baseline) (results not shown), the findings for MMSE changes should be interpreted more carefully.

Despite the limitations, there are several strengths of this study. Most of all, we used a nationally representative panel survey, which enables us to investigate a causal relationship between weight status to cognitive impairment/decline. To our knowledge, this is the first study that provides statistically reliable and robust evidence for an association between BMI with cognitive performance among middle- and old-age population in the region of Asia where the rate of aging-related diseases is rapidly increasing. In addition, we employed two outcome measures, both new-onset of severe cognitive impairment and cognitive decline over the follow-up, which provided consistent findings. Furthermore, we excluded individuals with mild to severe cognitive impairment (K-MMSE≤23) at baseline, and adjusted for a wide range of potential confounders including health-related behaviors and health status such as drinking, smoking, physical activity, activities of daily livings, depression, and comorbidities. Therefore, the findings could be robust toward potentially resolving the issue of reverse causation. Moreover, this study conducted subgroup analyses, which provide evidence for heterogeneity by a different socio-demographic characteristic. For example, we found the protective association of being obese with cognitive function is stronger among the worse-off.

In conclusion, our results suggest that being obese, especially in late-life, is related to lower risk of cognitive impairment, compared with having a normal weight. The reason for this inverse association is unclear at present. Many different issues—such as diet, frailty, and body composition change—could play a role. It is plausible that the role of BMI in dementia may change over the course of life. Further investigation is needed to understand the mechanism and the public health consequences of these findings.
